# Stabilization of the (C_2_H_5_)_4_NHSO_4_ High-Temperature Phase in New Silica-Based Nanocomposite Systems

**DOI:** 10.3390/molecules27248805

**Published:** 2022-12-12

**Authors:** Valentina Ponomareva, Irina Bagryantseva, Daria Dormidonova, Nikolai Uvarov

**Affiliations:** Institute of Solid State Chemistry and Mechanochemistry SB RAS, Kutateladze str. 18, 630090 Novosibirsk, Russia

**Keywords:** (C_2_H_5_)_4_NHSO_4_, organic acid salt, nanocomposites, solid electrolytes, proton conductivity

## Abstract

In this study, the electrotransport, thermal and structural properties of composite solid electrolytes based on (C_2_H_5_)_4_NHSO_4_ plastic phase and silica (1 − x)Et_4_NHSO_4−_xSiO_2_, where x = 0.3–0.9) were investigated for the first time. The composites were prepared by mechanical mixing of silica (300 m^2^/g, R_pore_ = 70Å) and salt with subsequent heating at temperatures near the Et_4_NHSO_4_ melting point. Heterogeneous doping is shown to change markedly the thermodynamic and structural parameters of the salt. It is important that, with an increase in the proportion of silica in the composites, the high-temperature disordered I4_1_/acd phase is stabilized at room temperature, as this determines the properties of the system. Et_4_NHSO_4_ amorphization was also observed in the nanocomposites, with an increase in the matrix contents. The enthalpies of the endoeffects of salt melting and phase transitions (160 °C) changed more significantly than the Et_4_NHSO_4_ contents in the composites and completely disappeared at x = 0.9. The dependence of proton conductivity on the mole fraction reached a maximum at x = 0.8, which was three or four orders of magnitude higher than the value for pure Et_4_NHSO_4_, depending on the composition and the temperature. The maximum conductivity values were close to those for complete pore filling. The conductivity of the 0.2Et_4_NHSO_4_-0.8SiO_2_ composite reached 7 ∗ 10^−3^ S/cm at 220 °C and 10^−4^ S/cm at 110 °C.

## 1. Introduction

One of the new, significant and interesting ways of improving the conductivity of ionic salts is heterogeneous doping with highly dispersed inert materials, for example, metal oxides with a high specific surface area and a large pore size [1,2,3]. The conductivity of composite systems can increase by several orders of magnitude due to surface interactions between the components, which lead to the formation of high defect concentrations in the areas of interfacial contact in composites (the so-called space charge regions) [4]. In some cases, especially in nanocomposites, changes in the thermodynamic parameters of ionic salts are observed [5,6]. This has been observed in a number of nanocomposites based on silica, zirconium, titanium dioxides and acid salts of alkali metals, as well as lithium salts [7,8,9,10,11,12].

For a number of acid salts of alkali metals, as a rule, having superionic phases, the possibility of creating highly conductive proton composite electrolytes has been shown [13,14,15]. In [16,17], composites based on CsHSO_4_ and highly dispersed silicon dioxide (1 − x)CsHSO_4−_xSiO_2_ with different specific surface areas and pore sizes were studied. It was shown that the conductivity of the low-temperature phase of CsHSO_4_ in the composite increased by up to 2.5 orders of magnitude, depending on the composition and type of silicon dioxide, and passed through a maximum. At the same time, decreases in temperature and the enthalpies of phase transitions and melting were observed, which were associated with the dispersion and amorphization of the salt. The largest size effect was obtained for composites based on SiO_2_ with a specific surface area of ~300–500 m^2^/g, which had a uniformly porous structure and an average pore radius of 35–70 Å [16,17,18]. The introduction of a dispersed matrix resulted in an increase in the conductivity of the low-temperature phase of the salt and a decrease in the high-temperature region due to the effect of conductor–insulator percolation. Thus, the properties of the composites were significantly affected not only by the types of heterogeneous components and the chemical natures of the surfaces, but also by the features of the porous structures of the matrices, in particular, the sizes of grains and pores and the nature of the pore size distribution.

**Figure 1:**
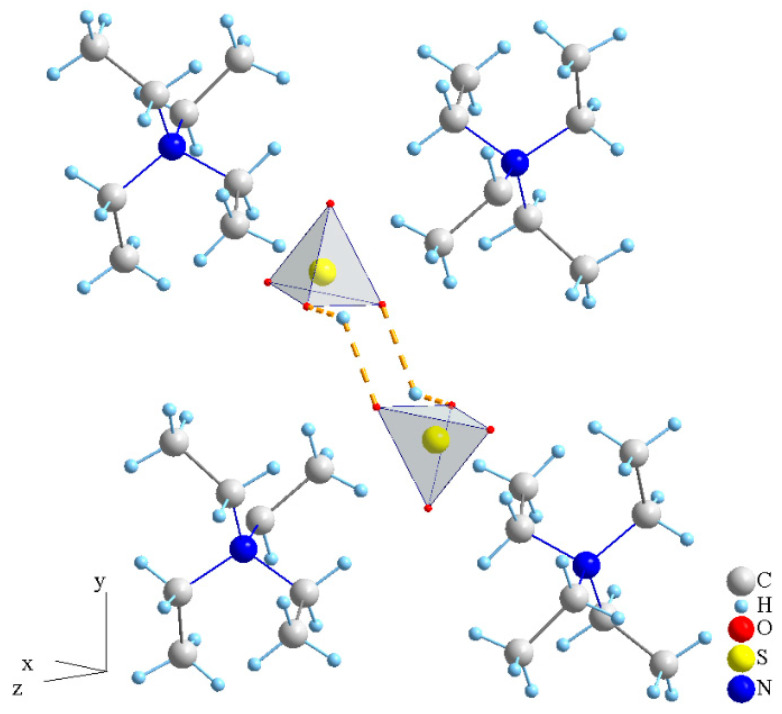
Schematic presentation of Et_4_NHSO_4_ crystal structure according to [26].

As for organic plastic crystals, which have attracted the interest of researchers in recent years [19,20], there are no data in the literature on composite systems based on them. The class of quaternary ammonium salts, which have the general formula R_4_N^+^X (R-alkyl, aryl group), consisting of both polar (N^+^) and non-polar (R) groups, exhibit a variety of physicochemical properties. Some of the quaternary ammonium compounds have ferroelectric properties, many of them are ionic liquids and parts of them are constituted by organic plastic crystals [21,22,23]. Accordingly, they are distinguished by a wide range of applications, being used as reagents, surfactants, catalysts, ionic liquids and electrolytes. As for the subgroup of quaternary ammonium hydrogen sulfates, such as methyl- (Me), -ethyl (Et), -propyl (Pr) and -butyl (Bu) ammonium, their structural, thermodynamic and electrotransport properties have not been studied enough. Recently, data have appeared on the structural properties and proton conductivities of Bu_4_NHSO_4_ [24,25] and Et_4_NHSO_4_ [26,27] compounds. As a rule, an increase in the length of the carbon chain of an alkyl group leads to a decrease in the melting temperature and a change in the symmetry of the unit cell from orthorhombic to monoclinic. The Et_4_NHSO_4_ compound is characterized by a rather high melting point due to the salts in this family of 245 °C [26]. At room temperature, Et_4_NHSO_4_ belongs to the monoclinic (P2_1_/n) system with the unit cell parameters a = 9.7994 Å, b = 13.812 Å, c = 9.5968 Å and β = 89.368° [26]. In the crystal structure of the salt, layers of tetraethylammonium cations alternate with anionic hydrosulfate layers (Figure 1).

Et_4_N^+^ cations have the shape of a Nordic cross with S_4_ symmetry and are much larger than the anions. The symmetry of SO_4_ groups is close to tetrahedral, with small deviations in the O-S-O angle from 109.28° for an ideal tetrahedron. Two sulfate tetrahedra are linked into dimers by strong hydrogen bonds. The dimers are ordered into layers that are isolated from each other at a relatively large distance of 6.53 Å [26]. At T = 160 °C, the tetragonal phase I4_1_/acd is formed with the unit cell parameters a = 14.0430 Å and c = 25.686 Å and exists up to the melting point of Et_4_NHSO_4_ [27].

The proton conductivity of Et_4_NHSO_4_ is quite low and does not exceed 5·10^−6^ S/cm at temperatures below 200 °C, which is due to the structural features of the compound and the presence of strong hydrogen bonds. The activation energy of conductivity varies non-linearly with temperature from 1.2 eV up to ~140 °C to 0.58 eV in the 140–200 °C temperature range. Further, a sharp increase in conductivity can be observed due to the achievement of temperatures close to the compound’s premelting temperature. The nature of the temperature dependence of the conductivity corresponds to the structural phase transitions of Et_4_NHSO_4_. A sequence of reversible phase transitions was established at 147 °C and 160 °C with enthalpies of −3.28 and −8.99 J/g, respectively [24,27]. Given the large size of Et_4_N^+^, the cationic subsystem constitutes the less mobile core of the crystal lattice. The most probable charge carriers are protons associated with SO_4_ tetrahedra.

This study aimed to synthesize proton composite electrolytes of (1 − x)Et_4_NHSO_4−_xSiO_2_ (where x = the mole fraction of silica) with a wide range of compositions and investigate their electrotransport and structural properties. Silicon dioxide with a specific surface area of 300 m^2^/g, a pore size of 70 Å with a uniform pore size distribution and a pore volume of 0.9 cm^3^/g was used as a matrix, which, as was shown earlier, was characterized by the optimal morphology for the composites based on the acid salts of alkali metals [28].

## 2. Materials and Methods

Composite electrolytes of (1 − x)Et_4_NHSO_4−_xSiO_2_, in which the molar fraction of SiO_2_, *x*, varied from 0 to 0.90, were prepared. Commercial reagent Et_4_NHSO_4_ of analytical grade (Sigma-Aldrich) was used. Silicon dioxide samples with the trademark “KSKG” were preliminarily heated in air at 500 °C for 3 h. The porous structure of silica was analyzed using the nitrogen adsorption technique on a Quantochrome AutosorbiQ gas sorption analyzer at 77 K. Then, SiO_2_ was carefully ground in an agate mortar with tetraethylammonium hydrogen sulfate, at a certain molar ratio. The molar fractions were selected following various degrees of pore filling, including the complete filling of SiO_2_ pores with salt. The homogenized powder mixture was pressed at P = 500 MPa with the application of silver or platinum electrodes. The resulting tablets were heated in air for 20 min at a temperature (T) = 220–230 °C, close to the melting point of the salt. The electrical conductivity was measured with a two-electrode circuit and an alternating current using an IPU-1RLC-1/2008 impedance meter (frequency range: 0.1 Hz–3.3 MHz) in a temperature range of 25–250 °C by the complex impedance method. The measurements were carried out in the slow cooling mode at a rate of 0.5–1 deg/min in air, with a relative humidity of 15–20%.

The phase composition of the (1 − x)Et_4_NHSO_4−_xSiO_2_ electrolytes was analyzed by X-ray diffraction with a D8 Advance diffractometer (Cu-Kα radiation). Fourier transform infrared spectroscopy (FTIR) spectra in the attenuated total reflectance (ATR) mode were recorded on a Bruker Tensor 27 spectrometer. The morphology of the samples was investigated using a Hitachi S3400N scanning electron microscope. Differential scanning calorimetry (DSC) data were obtained with a DSC 500 differential calorimeter in heating mode in the temperature range of 25–260 °C with an argon flow at a rate of 10 °C/min.

## 3. Results and Discussion

**Figure 2:**
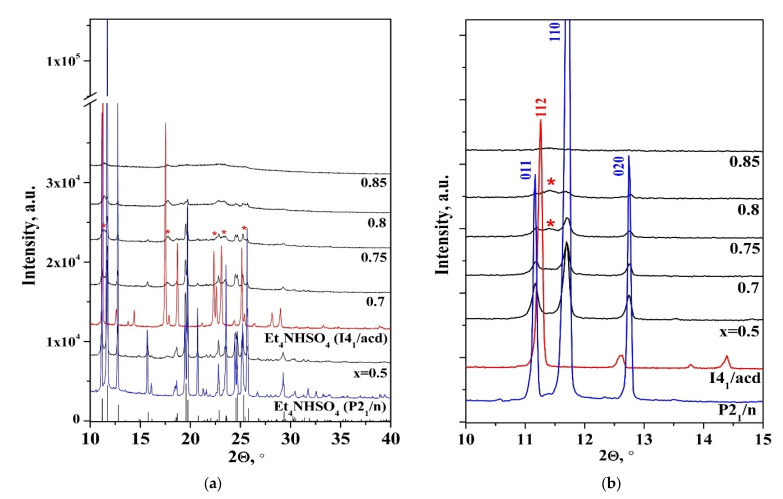
PXRD patterns of (1 − x)Et_4_NHSO_4−_xSiO_2_ synthesized composites with different compositions at room temperature. Vertical markers indicate the calculated positions of Bragg reflections of Et_4_NHSO_4_; asterisks indicate the reflections of Et_4_NHSO_4_ in the I4_1_/acd phase. The PXRD patterns are presented for 2θ ranges of 10–40 (**a**) and 10–15 (**b**) degrees.

The powder X-ray diffraction (PXRD) patterns of the synthesized (1 − x)Et_4_NHSO_4−_xSiO_2_ composites at room temperature are presented in Figure 2. The PXRD pattern of Et_4_NHSO_4_ (P2_1_/n) at room temperature completely coincided with the results of [26]. The intensity of reflexes of the composites decreased significantly, and the peaks broadened with the composition x = 0.5. It was clear that at x = 0.5 all reflections corresponded to the Et_4_NHSO_4_ (P2_1_/n) crystal structure (Figure 2a). Therefore, chemical interaction between the salt and the dispersed SiO_2_ matrix in the composite could be excluded. The intensity of reflexes decreased more significantly than the mass fraction of Et_4_NHSO_4_ in the composite compounds. A more detailed view of the main intense reflections is shown in Figure 2b. For example, the intensity of some of the peaks in the monoclinic phase (011, 110, 020) decreased more than 10 times for the x = 0.5 composition, while the salt content in the composite was 79 w%; at x = 0.7, the intensity decreased up to 35 times when the salt content in the composite was equal to 62 w% (Figure 2b). With a further increase in the heterogeneous matrix content up to x = 0.7, the reflections of the high-temperature I4_1_/acd phase appeared; they are indicated by asterisks in Figure 2a. In a number of intermediate compositions, the existence of two phases of Et_4_NHSO_4_, both low-temperature (P2_1_/n) and high-temperature (I4_1_/acd), was observed. 

At x > 0.8, the PXRD patterns showed that the salt in the composite was mainly in the high-temperature I4_1_/acd phase with a significantly reduced intensity of reflections and their conspicuous broadening. This was due to the formation of Et_4_NHSO_4_ in the amorphous state on the highly dispersed SiO_2_ surface and pores. At x > 0.85, the reflexes almost disappeared. In addition, the reflexes of Et_4_NHSO_4_ in the composites moved towards larger angles due to a slight decrease in the Et_4_NHSO_4_ unit cell parameters on the surface of the silica (Figure 2b). Thus, the PXRD patterns showed that the salt was retained in the composites with the crystal structure of Et_4_NHSO_4_ (P2_1_/n) at lower silica additive levels (up to x = 0.5). With further increase in x, two disordered Et_4_NHSO_4_ phases with decreased unit cell parameters existed in the composites, and then, with an increase in x, at x ≥ 0.8 the high-temperature phase (I4_1_/acd) stabilized and existed in the composites at a low temperature. It is remarkable that almost the same changes were observed in the FTIR spectrum for CsHSO_4_ [15], indicating that in the composite with highly dispersed silicon dioxide (Ssp ~300 m^2^/g) CsHSO_4_ was stabilized in the more disordered phase II (P2_1_/c) with weaker hydrogen bonds, or, depending on the composition, there was a mixture of phases III (P2_1_/m) and II, with phase II being the most prevalent.

**Figure 3:**
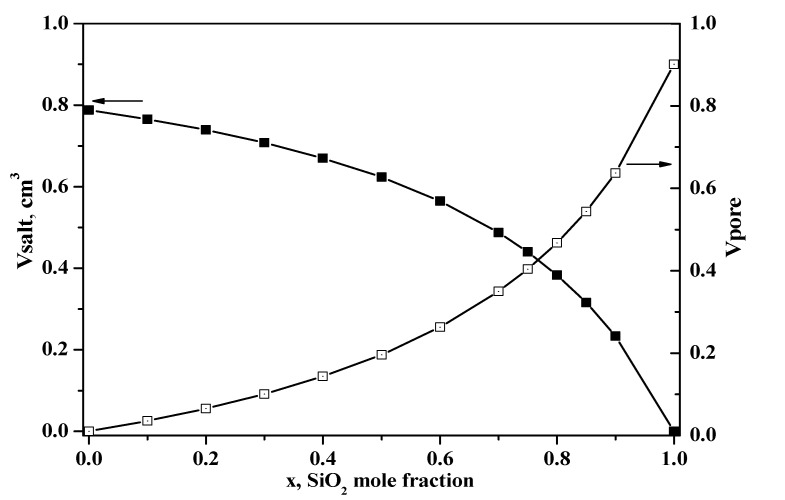
The variation in the total volume of Et_4_NHSO_4_ (V_salt_) and the total volume of silica pores (V_pore_) per 1 g of the composite with the SiO_2_ mole fraction in the (1 − x)Et_4_NHSO_4−_xSiO_2_ composites.

For a more detailed investigation of the change in structural, thermodynamic and electrotransport properties, we chose the compositions corresponding to various degrees of pore filling, including an excess or deficiency of salt relative to the available pore volume. The dependences of the volume of Et_4_NHSO_4_ (V_salt_) and the total pore volume of silica (V_pore_) per 1 g of composite as a function of the mole fraction, x, are shown in Figure 3. As discussed below, the salt content exceeded the pore volume in the composites up to x = 0.8. The complete filling of the pores could be observed at x = 0.8 in the case of an ideal distribution of components. At x > 0.8, the volume of the matrix pores exceeded markedly the volume of the salt, which can lead to incomplete pore filling and a significant decrease in conductivity, due to disruption of the formed conduction channels. Therefore, the compositions close to the complete filling of pores were studied in more detail.

**Figure 4:**
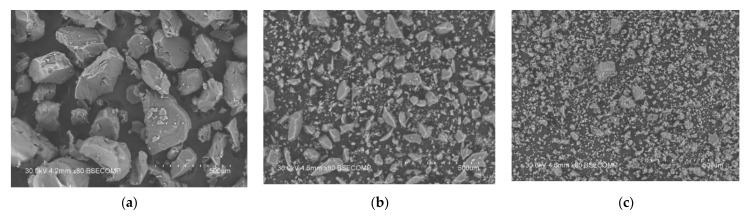
Scanning electron microscopy images of pure Et_4_NHSO_4_ (**a**), silica (**b**) and the composite 0.2Et_4_NHSO_4_-0.8SiO_2_ powders (**c**) represented at the same magnification.

The pore-filling effect was supported by the results of the scanning electron microscopy studies presented in Figure 4. As can be seen from the figure, the initial Et_4_NHSO_4_ powder consisted of coarse crystalline particles 100–300 μm in size (Figure 4a).

Pure silica powder is characterized by a wide particle size distribution, with the particle size ranging from 5 to 100 μm (Figure 4b). The morphology of the 0.2Et_4_NHSO_4_-0.8SiO_2_ composite (Figure 4c) is similar to that for pure silica. The disappearance of large crystallites of Et_4_NHSO_4_ may be explained by the imparting of the salt into the pores of the silica, the morphology of the silica particles being unaffected.

**Figure 5:**
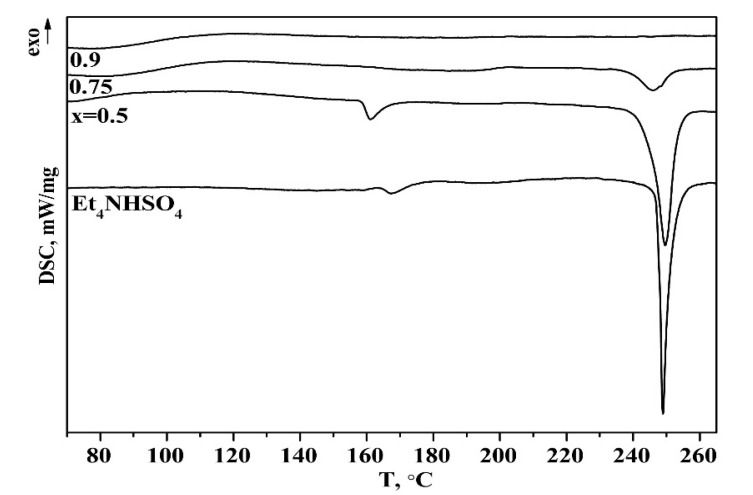
DSC data for Et_4_NHSO_4_ and (1 − x)Et_4_NHSO_4−_xSiO_2_ composites with different compositions.

DSC data for Et_4_NHSO_4_ and the (1 − x)Et_4_NHSO_4−_xSiO_2_ composites are presented in Figure 5. It can be seen that the enthalpies of the endoeffects of the melting and phase transitions changed more significantly than the salt content in the composites and completely disappeared with an increase in x to 0.9. The temperature and enthalpy of melting for the composite when x = 0.5 were closer to the original salt (244.8 °C and -71.97 J/g, accordingly) and the enthalpy for x = 0.75 decreased markedly and was equal to -14.8 J/g. The temperature of melting shifted to 239 °C. The temperature of the phase transition also decreased to 158.7 °C. With the further increase in x to x = 0.9, the endoeffects due to the melting and phase transitions disappeared.

**Figure 6:**
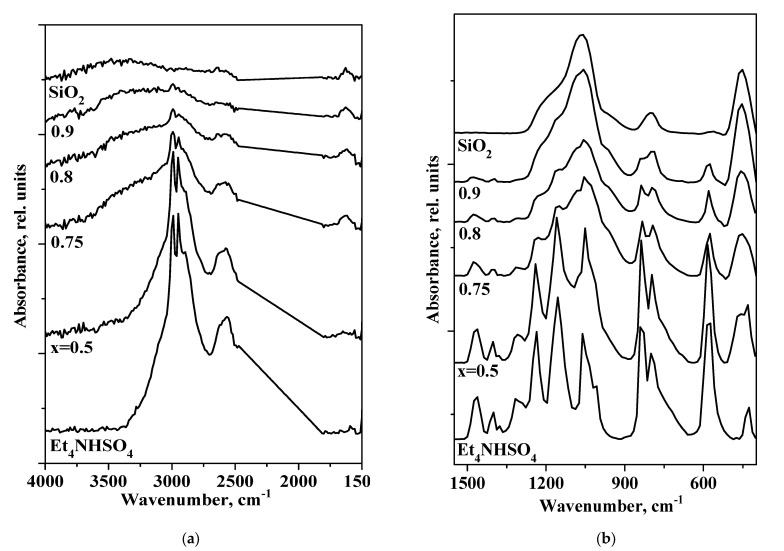
FTIR spectra of Et_4_NHSO_4_, SiO_2_ and (1−x)Et_4_NHSO_4−_xSiO_2_ composites of different compositions presented in different spectral ranges: hydrogen bond network (**a**) and sulfate groups (**b**).

FTIR spectra for Et_4_NHSO_4_ are shown in Figure 6 for hydrogen bond networks (a) and SO_4_ tetrahedra and Et_4_N^+^(b). The wavenumbers of the stretching and bending vibrations of the SO_4_ tetrahedra in Et_4_NHSO_4_ correspond to inorganic hydrogen sulfate data [29,30] and other salts with organic cations [31,32,33]. There were intense absorption bands at 3500–1500 cm^−1^, corresponding to the strong hydrogen bond network, and ~1243 cm^−1^, characteristic of acid sulfates. The decreased symmetry of the T_d_ for SO_4_^2^-containing compounds to C_3v_ symmetry for HSO_4_^−^ resulted in the splitting of the characteristic tetrahedral bands. Since the symmetrical stretching vibrations of Et_4_N^+^ extended into the area of absorption bands of the sulfate ion, the spectrum was less easily interpreted. The characteristic ABC structure, observed for strong hydrogen bonds of Et_4_NHSO_4_, should be explained based on Fermi resonance between the OH stretching, the overtone of the out-of-plane bending vibrations 2γ(OH) and the combination of the in-plane bending δ(OH) mode and the out-of-plane bending vibrations γ(OH), which give rise to the characteristic doublet with maxima at ca. 2941 cm^−1^ (A), 2607–2534 cm^−1^ (B) and the broad, weakly expressed band at ca 1630 cm^−1^ (C). The in-plane bending mode (δ_OH_) of the hydrogen bond gave a strong band at 1236 cm^−1^. A more detailed correlation of absorption bands for an organic cation is presented in [27,34,35].

**Figure 7:**
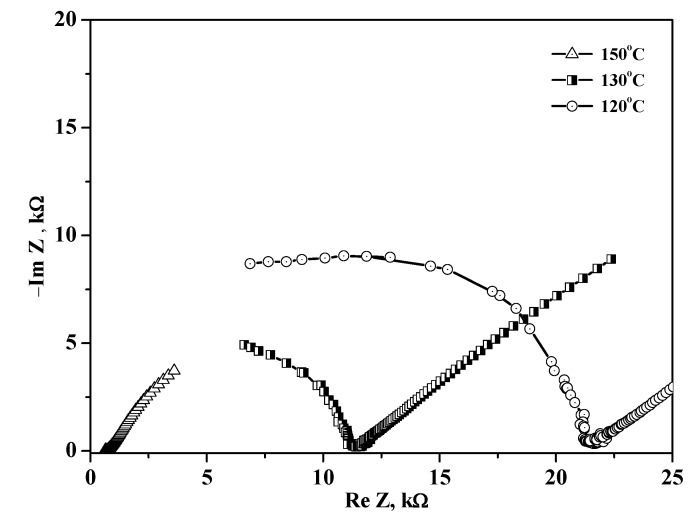
The impedance spectra of the (1 – x)Et_4_NHSO_4−_xSiO_2_ composites (x = 0.8) measured at different temperatures.

The FTIR spectrum of the Et_4_NHSO_4_-SiO_2_ composite can be interpreted as the sum of the spectra for SiO_2_ and Et_4_NHSO_4_. In the Et_4_NHSO_4_–SiO_2_ composites, the intensity of the absorption bands in the region of hydrogen bonds decreased significantly and the centers of gravity of the corresponding absorption bands slightly shifted to the higher frequencies. The a.b in the range of 2564 cm^−1^ shifted to 2573 cm^−1^ at x = 0.5 and to 2583 cm^−1^ at x = 0.8, while the center of gravity of the silica absorption bands of 3442 cm^−1^ shifted to 3353 cm^−1^ at x = 0.9 due to the formation of weak hydrogen bonds between the salt and the matrix. The changes were also observed in the spectral range of the sulfate group: the absorption bands decreased markedly in intensity for x > 0.75 and the a.b. in the range of 1148 cm^−1^ shifted to 1158 cm^−1^, while the a.b of 802 cm^−1^ shifted to 790 cm^−1^ for x > 0.75. The changes were associated with a slight strengthening of the S–O bonds and a weakening of the hydrogen bond network of the salt upon binding to the silica matrix. The differences between the FTIR spectrum of the composite at x = 0.8 and the spectrum of Et_4_NHSO_4_ were more pronounced. There was a shift—a significant decrease in the intensity of the absorption bands—corresponding to the stretching and bending vibrations of SO_4_ and their relative broadening, which corresponded to an increase in the symmetry of SO_4_ tetrahedra and an increase in the orientational disorder of HSO_4_^-^ ions.The impedance plots of the composites (x = 0.8) at different temperatures are presented in Figure 7. The Nyquist plot (showing the dependence of imaginary and real parts of resistivity) consists of an arc representing the electrode processes (in the region of lower frequencies) and parts of semicircles due to electrolyte transfer in the higher frequency region. The proton conductivity was calculated from the resistance values with the minimum of capacitive components. With increasing temperature, the radii of the semicircles decreased markedly, so the proton conductivity increased due to an increase in the number of current carriers and their mobility.

**Figure 8:**
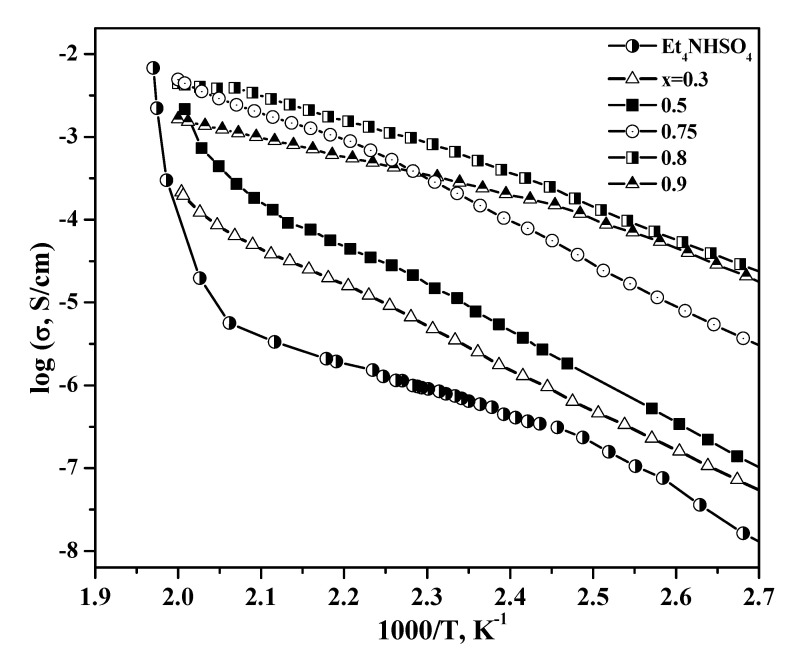
Temperature dependencies of conductivity of Et_4_NHSO_4_ and (1 – x)Et_4_NHSO_4_–xSiO_2_ with different compositions, x = 0.5–0.9 (cooling regime, air, 0.5–1 deg/min).

The temperature dependencies of the conductivity for (1 – x)Et_4_NHSO_4_–xSiO_2_ in comparison with the original salt are presented in Figure 8. The low-temperature conductivity of Et_4_NHSO_4_ obeys the Arrhenius law, with an activation energy of ~1.2 eV. The conductivity of the Et_4_NHSO_4_ was rather low and did not exceed 5·10^−6^ S/cm at temperatures below 210 °C, which corresponded to the structural data and the strong hydrogen bond network of the compound. The activation energy of conductivity varied with the temperature from 1.2 eV up to ~140 °C and then showed a linear region at 140–200 °C with the activation energy of 0.58 eV.

A significant increase in conductivity by three orders of magnitude for Et_4_NHSO_4_ was observed at temperatures above 210 °C, close to the melting point, where the conductivity reached ~8·10^−3^ S/cm, characteristic of the melts. Heterogeneous doping of Et_4_NHSO_4_ resulted in a significant increase in the conductivity by three or four orders of magnitude, depending on the composition and temperature. The conductivity increased by up to one order of magnitude in the investigated composites (with small x values up to x = 0.5), which may be connected to the dispersion of salt and the formation of space charge regions at the interphase with the amorphous state of Et_4_NHSO_4_ on the highly dispersed SiO_2_ surface (x > 0.5) and in pore spaces. Two regions of temperature dependence of the conductivity could also be distinguished for the composite Et_4_NHSO_4_–SiO_2_ systems with a change in the activation energy. At temperatures lower than 170 °C, the activation energy was equal to 1.1 eV for x = 0.5, decreased to 1.0 eV for x = 0.75 and was equal to 0.76 eV for x = 0.8. A decrease in the activation energy with an increase in the inert highly dispersed additive is typical for composite solid electrolytes and is described by partial salt amorphization and the contribution of the interfaces to the overall conductivity, the number of which increases with x [17,36]. At higher temperatures, >170 °C, close to the phase transition to I4_1_/acd phase, the activation energy changed to 0.45 eV for x = 0.75–0.9. The maximum conductivity reached high values of ~7·10^−2^ S/cm at 220 °C for x = 0.8. This composition corresponded to the complete filling of pores (Figure 3) and was characterized by high conductivity values in low and high temperature ranges. This indicated high values of salt adhesion and meant that the selected highly dispersed silica matrix with the pore radius of 70 Å and uniform pore size distribution was optimal for the Et_4_NHSO_4_. In this case, not only did salt dispersion, disordering and amorphization occur; the high-temperature I4_1_/acd phase was also stabilized at low temperatures. The conductivity values for the studied systems are some of the highest among acid hydrogen sulfate composites [16]. Such size effects have been observed for a number of compounds. Phenomena of size effect, in which the dispersion of salts in the fine pores of an inert component (r_pore_ < 100 Å) leads to qualitative changes in their bulk structure, including the appearance of high-temperature modifications, partial or complete amorphization and change in the number of thermodynamic characteristics, such as the enthalpies of the melting and phase transitions, and temperature, have been shown in previous studies [16,17,18,37,38,39,40,41]. For example, in the case of the SiO_2_ matrix with a small pore radius (40 Å), only the high-temperature modification of KNO_3_ and the amorphous CsCl phase were detected, which, according to the authors, were stable at room temperature for a long time.

**Figure 9:**
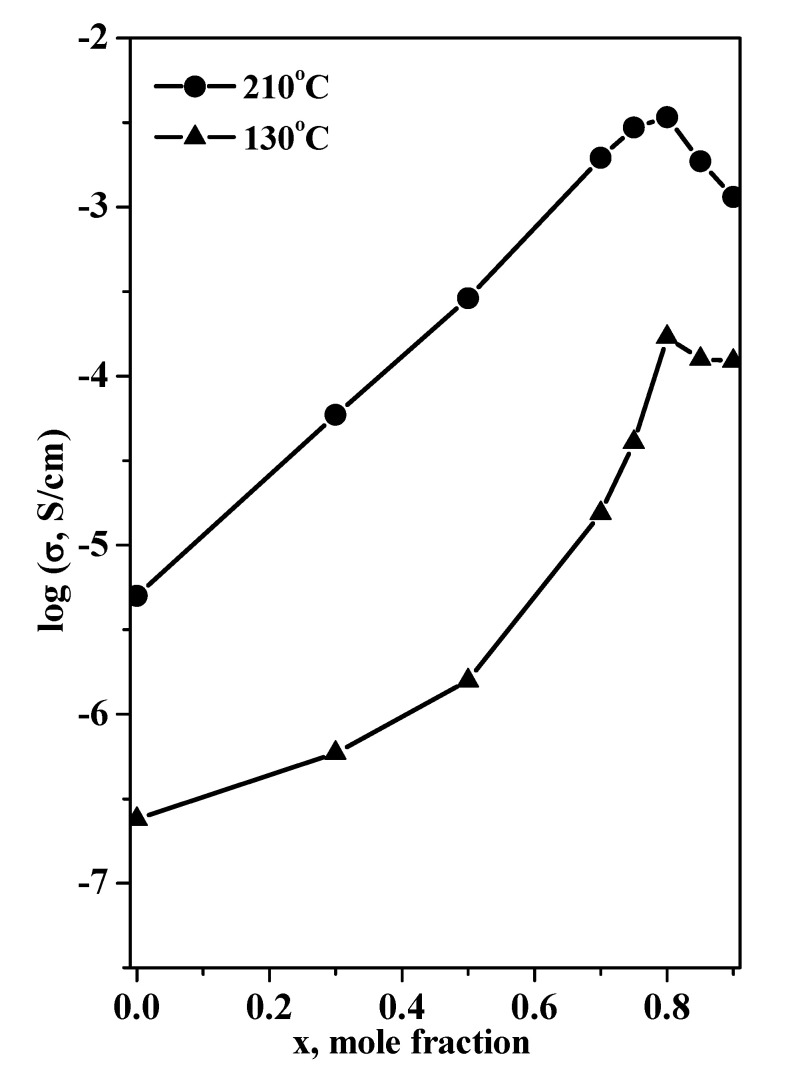
The isotherms of the conductivity of (1 – x)Et_4_NHSO_4_–xSiO_2_ at different temperatures as a function of the mole fraction of SiO_2_.

Figure 9 shows isotherms of conductivity as a function of the mole fraction of silica for different temperatures.

The dependence of conductivity on the mole fraction reached a maximum, with a value up to three to four orders of magnitude higher than that of the pure Et_4_NHSO_4_, depending on composition and temperature. The conductivity reached a maximum at x = 0.8 at lower and higher temperatures, which was close to that for the filling of pores. At higher silica contents (x > 0.8), the pore volume exceeded markedly the total volume occupied by the salt, and the conductivity decreased due to the conductor–insulator percolation effect. A significant size effect for the heterogeneous matrix influence on the electrotransport, structural and thermodynamic properties for plastic tetraalkyl ammonium hydrosulfate crystals was obtained for the first time. The effect was associated with the optimal morphology of silica with a pore size of 70 Å and a high energy of adhesion. Further research is important for more detailed elucidation of the mechanism of the formation of nanocomposites and their conductivity.

## 4. Conclusions

Composite solid electrolytes based on plastic tetraalkyl ammonium crystals and silica (1 − x)Et_4_NHSO_4−_xSiO2 (x = 0.3–0.9) in a wide range of compositions were synthesized and investigated for the first time. The heterogeneous doping with silica changed markedly the thermodynamic, structural and electrotransport parameters of the salt. The morphology, pore size and surface nature of the silica were optimal for the introduction of Et_4_NHSO_4_, which determined the properties of the salt in the nanocomposites. Compositions close to the complete filling of pores were studied in more detail. Significant structural changes in the phase composition of Et_4_NHSO_4_ in the composites were found, depending on the silica mole fraction and the degree of filling of the pore space of the matrix. The existence of two disordered phases, the original low-temperature (P2_1_/n) and high-temperature (I4_1_/acd) phases of Et_4_NHSO_4_, was observed under normal conditions in the nanocomposites. With increase in the silica mole fraction, the content of the high-temperature phase increased. At x = 0.8, which corresponds to the complete filling of the pores, the Et_4_NHSO_4_ salt in the composites was mainly in the disordered high-temperature I4_1_/acd phase. A slight decrease in the Et_4_NHSO_4_ unit cell parameters in the silica pores was observed. The thermodynamic, structural and electrotransport properties were in full agreement. The enthalpies of endoeffects of salt melting and phase transitions (160 °C) changed more significantly than the salt content of Et_4_NHSO_4_ in the composites and completely disappeared with x = 0.9 due to salt amorphization. The intensity of absorption bands corresponding to P-O and hydrogen bond networks decreased significantly with x content, and the a.b. slightly shifted to higher frequencies as a result of the formation of weak hydrogen bonds between the salt and the matrix. The dependence of proton conductivity on mole fraction reached a maximum at x = 0.8, with the value up to three or four orders of magnitude higher than that of pure Et_4_NHSO_4_, depending on the composition and the temperature, which was close to that for complete pore filling. The conductivity of the 0.2Et_4_NHSO_4_-0.8SiO2 composite reached 7 ∗ 10^−3^ S/cm at 220 °C. A significant size effect of the influence of the heterogeneous matrix on the electrotransport, structural and thermodynamic properties of plastic tetraalkyl ammonium crystals was obtained for the first time and was due to the optimal morphology of silica with a pore size of 70Å and the energy of adhesion.

## Data Availability

Not applicable.

## Sample Availability

Samples of the (1 − x)Et_4_NHSO_4−_xSiO2 (x = 0–0.9) composites are available from the authors.
